# Serum protein biomarkers for HCC risk prediction in HIV/HBV co-infected people: a clinical proteomic study using mass spectrometry

**DOI:** 10.3389/fimmu.2023.1282469

**Published:** 2023-11-10

**Authors:** Hengning Ke, Rui Yuan, Huan Liu, Mingqi Luo, Hui Hu, Ejuan Zhang, Ke Zhuang, Yong Yang, Rongrong Yang

**Affiliations:** ^1^ Department of Infectious Diseases, Zhongnan Hospital of Wuhan University, Wuhan, Hubei, China; ^2^ Center for AIDS Research, Wuhan University, Wuhan, Hubei, China; ^3^ Medical Science Research Center, Zhongnan Hospital of Wuhan University, Wuhan, China; ^4^ Animal Biosafety Level 3 Laboratory at the Center for Animal Experiment, State Key Laboratory of Virology, Wuhan University, Wuhan, Hubei, China; ^5^ SpecAlly Life Technology Co., Ltd., Wuhan Institute of Biotechnology, Wuhan, China

**Keywords:** hepatocellular carcinoma, human immunodeficiency virus, hepatitis B, golgi-associated plant pathogenesis-related protein 1, phospholipid transfer protein

## Abstract

**Background:**

HBV coinfection is frequent in people living with HIV (PLWH) and is the leading cause of hepatocellular carcinoma (HCC). While risk prediction methods for HCC in patients with HBV monoinfection have been proposed, suitable biomarkers for early diagnosis of HCC in PLWH remain uncommon.

**Methods:**

Liquid chromatography-tandem mass spectrometry (LC-MS/MS) was used to examine serum protein alterations in HCC and non-HCC patients with HIV and HBV co-infection. Gene Ontology (GO), Kyoto Encyclopedia of Genes and Genomes (KEGG), and Disease Ontology (DO) enrichment analysis were performed on the differentially expressed proteins (DEPs). The risk prediction model was created using five-cross-validation and LASSO regression to filter core DEPs.

**Results:**

A total of 124 DEPs were discovered, with 95 proteins up-regulated and 29 proteins down-regulated. Extracellular matrix organization and membrane component were the DEPs that were most abundant in the categories of biological processes (BP) and cellular components (CC). Proteoglycans in cancer were one of the top three DEPs primarily enriched in the KEGG pathway, and 60.0% of DEPs were linked to various neoplasms in terms of DO enrichment. Eleven proteins, including GAPR1, PLTP, CLASP2, IGHV1-69D, IGLV5-45, A2M, VNN1, KLK11, ANPEP, DPP4 and HYI, were chosen as the core DEPs, and a nomogram was created to predict HCC risk.

**Conclusion:**

In HIV/HBV patients with HCC, several differential proteins can be detected in plasma by mass spectrometry, which can be used as screening markers for early diagnosis and risk prediction of HCC. Monitoring protease expression differences can help in the diagnosis and prognosis of HCC.

## Introduction

Hepatocellular carcinoma (HCC) is the sixth most common cancer and the second leading cause of cancer-related deaths worldwide, killing over 700,000 people each year (1, 2). In contrast to most solid tumors, the prevalence of HCC and HCC-related mortality has risen in recent decades. Chronic hepatitis B virus (HBV) and chronic hepatitis C virus (HCV) infection are the most important causes of HCC. Overall, HCC occurs lifelong in 15% to 40% of chronic HBsAg carriers in the absence of antiviral treatment (3). Human immunodeficiency virus (HIV) is typically spread by sexual, parenteral, and neonatal transmission, which explains why HIV-HBV co-infection is relatively common. HBV infection among HIV-infected patients is estimated to be 5% to 15% (3, 4). Thus, HBV co-infection affects 3 million of the 35 million people living with HIV worldwide (3, 4). When compared to HBV-monoinfected individuals, HIV-HBV co-infected patients have a lower chance of spontaneous HBeAg and HBsAg clearance, and serum HBV-DNA levels are higher, which may contribute to a faster progression to end-stage liver disease and HCC, which is characteristic of HIV-HBV co-infected patients (5–7). As a result, HCC continues to be one of the leading causes of non-AIDS deaths among people living with HIV (PLWH) (8).

The traditional marker used for HCC risk monitoring is AFP (alpha-fetoprotein), although it has largely failed in the early diagnosis of HCC (9). Other alternative biomarkers have been proposed, such as AFP-L3 (Lens culinaris agglutinin-reactive fraction of alpha-fetoprotein or highly sensitive -hs-AFP-L3) (10) and PIVKA-II (Protein induced by vitamin K absence or antagonist-II) (11), but the levels of these two proteins typically increase as HCC develops and progresses to portal vein invasion (11). Other indicators, such as the Mac-2-binding protein glycosylation isomer (M2BPGi), have been used to predict hepatocarcinogenesis in patients receiving nucleoside analogs (12, 13). Some indexes, such as aMAP (14), PAGE-B index (15, 16), and GALAD score (17), developed from different statistical models, demonstrated superior specificity and predictive value for HCC evaluation and monitoring. Several novel predictive biomarkers for HCC, such as methylation status of HOXA1, TSPLY5, and PFKP from cell-free DNA (18, 19), and Telomerase Reverse Transcriptase (TERT) promoter mutations (C228T and C250T) (20), are frequently detected in HCC and have a good predictive value for hepatic oncogenesis. None of these biomarkers or scores, however, have been studied in HIV-HBV co-infected patients. Appropriate biomarkers for the early identification of HCC in patients with HIV and HBV co-infection are still lacking. There is a practical need to create non-invasive, cost-effective, and easy serological diagnostic markers for HCC early detection, particularly in those living with HIV and HBV co-infection in resource-limited settings.

Mass spectrometry (MS)-based proteomics is useful for identifying and measuring protein post-translational changes. The utility of this technique has improved dramatically in recent years. Many research have used mass spectrometry proteomics to screen and identify serum potential biomarkers for illness diagnosis and prognosis evaluation in a variety of diseases, which has played a significant role in promoting clinical practice. However, proteomics research in PLWH is currently limited.

This study focused on HIV/HBV co-infected individuals who were separated into HCC and non-HCC groups based on their clinical diagnoses. First, we tried to screen out prospective biomarkers in the assessment of HCC by detecting the expression of differentially expressed proteins (DEPs) related to HCC. Then, we intend to provide new clues for the in-depth investigation of HCC pathogenesis from the standpoint of proteomics by doing a functional enrichment analysis of DEPs between HCC patients and non-HCC patients. Furthermore, we attempted to assess the probability of HCC development in individuals at high risk of developing end-stage liver disease by establishing an HCC risk prediction model.

## Methods

### Participants

**Figure 1:**
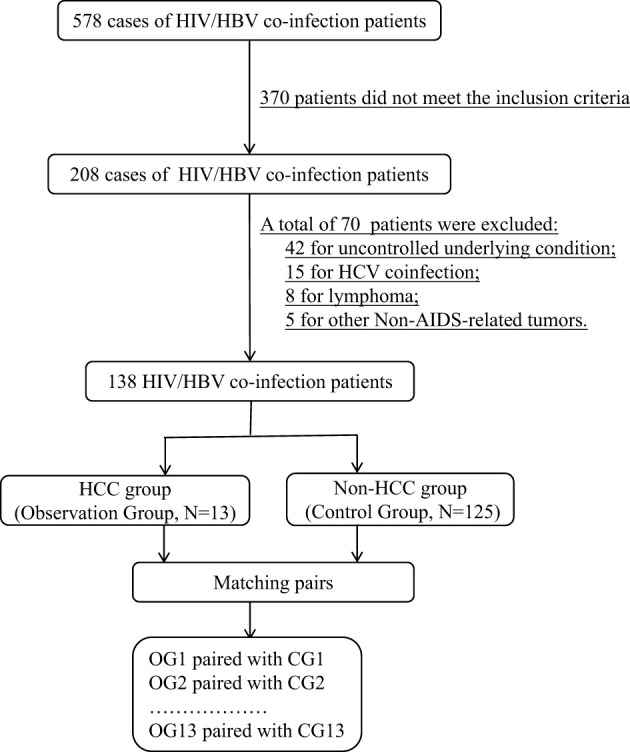
The flow chart of study object selection.

From 578 HIV/HBV co-infected patients, 13 pairs of HCC patients and non-HCC patients with matched information were chosen for the proteomic investigation. **Figure 1** depicts the flow chart of study object selection. The following were the inclusion criteria: (1) be 30-70 years old; (2) have complete HIV clinical data, including ART status and current CD4+ T lymphocyte counts; (3) have complete HBV clinical data, including hepatitis B indicators, HBV DNA levels, and liver imaging evaluation findings; and (4) have an HCC definition or exclusion diagnosis. Exclusion criteria included the following: (1) being pregnant or nursing; (2) an uncontrolled underlying condition, (3) organ dysfunction, (4) liver illnesses caused by other hepatitis virus infections, (5) any cancer other than HCC, and (6) diseases other than viral hepatitis that may cause HCC, such as metabolic problems, substance abuse, and genetic conditions.

For proteomic analysis, age, gender, clinical routine test indexes, CD4+ T lymphocyte counts, HBeAg status, liver function findings, duration of HBV infection, HIV and HBV replication levels, and other known characteristics influencing HCC progression had to be well matched. Following the fulfillment of all of the parameters for proteomic analysis, 26 patients with HIV and HBV coinfection who were successfully matched were divided into two groups for future proteome research: HCC (n=13) and non-HCC (n=13). The HCC patients were all in stage I.

This study was approved by the institutional review board of the Zhongnan Hospital of Wuhan University(2021022). An informed consent form was signed by all patients.

### Plasma sample preparation

Blood samples were collected from patients living with HIV and HBV co-infection between March 1, 2021 and December 31, 2022. For one-step reduction and alkylation, the samples were treated for 1 hour at 60°C with 1% SDC/100 mM Tris-HCL/10 mM TCEP/40 mM CAA (pH=8.5). SDC was diluted to less than 0.5% using ddH2O. Trypsin was added at a 1:50 (enzyme:protein, w/w) ratio for overnight digestion at 37°C. The following day, TFA was added to drop the pH to 6.0 and end the digestion. The supernatant was centrifuged for 15 minutes at 16000g before peptide purification using a self-made SDB desalting column. The peptide eluate was vacuum dried and stored at -20°C for later use.

### LC-MS/MS analysis

Tryptophan was dissolved in solvent A, which contained 0.1% formic acid. After that, the reverse-phase column is installed. The progressive gradient is constructed of solvent B containing 0.1% formic acid. Using the EASY-nLC system, the ascending gradient is filled with a mixture of formic acid concentrations ranging from 6% to 23%, and the flow rate is 400nL/min. The sample is subjected to a series of MS/MS assays on Thermo Scientific’s QExactiveTM Plus platform after the initial nano spray ionization process. The voltage of the electrospray is set to 2.0 kV. It is utilized for peptide activation and has a resolution of 70,000 to 1800. Make use of NCE Settings For MS/MS testing, peptides should be used. Orbitrap was then used to carry out a resolution. Data-dependent apps are executed dynamically on different scans. The fixed mass was then set to 100 m/z.

### Database search

Briefly, the raw MS data were analyzed with the DIA-NN program (V1.7.16). Tandem MS data were thoroughly referred to the SwissProt human protein sequences database. The deep learning algorithm in DIA-NN predicted a spectrum library, and the original DIA data were retrieved by the anticipated spectrum library and the spectrum library acquired by MBR function to obtain protein quantitative information. As a consequence, the parent ion and protein levels were tested using 1% FDR. Following screening, the quantitative information from the proteome was used for further analysis.

### Biological information analysis

The function databases employed in this work were Gene Ontology (www.geneontology.org) (GO), Kyoto Encyclopedia of Genes and Genomes (http://www.genome.jp/kegg/) (KEGG), and Disease Ontology (https://disease-ontology.org/) (DO). Annotation information for GO, KEGG, and DO was collected by mapping identified proteins to matching databases downloaded from the official website. Then, using CytoScape software and the “CytoHubba” plug-in, network maps of DEPs were created using protein-protein interaction (PPI) data from the STRING database, and the hub proteins were further filtered.

### Statistical analysis

In the descriptive statistical analysis of the study subjects’ demographic and clinical features, means and standard deviations were utilized for continuous variables, while proportions or rates were provided for categorical data. The fold change and P value less than 0.05 were chosen as the key screening criteria for biomarkers. The penalty term was chosen using five-fold cross-validation, and the parameter for LASSO regression analysis with the smallest model fitting error was utilized to filter the variables. The model was tested in three ways: differentiation using ROC area AUC, calibration and consistency using calibration curve, and clinical effect using DCA clinical decision curve analysis or nomogram. The R programming language was utilized for statistical analysis and model development.

## Results

### Demographic and clinical characteristics

**Table 1** shows the demographic and clinical features of the patients with HIV and HBV co-infection who participated in this investigation. In terms of age, gender, fraction of cART treatment, and anti-HBV therapy, no significant differences were found between the HCC and non-HCC groups. None of the clinical indicators, including HIV-RNA and HBV-DNA levels, HBeAg status, blood routine test results, and liver function test findings, were substantially different between the HCC and non-HCC groups.

**Table 1:** Demographic and clinical characteristics of people living with HIV and HBV co-infection in this study.

	People living with HIV/HBV co-infection		Statistical value	*P*-value
	HCC group (n=13)	Non-HCC group (n=13)		
Age, mean ± SD, years	48.7 ± 10.8	48.8 ± 12.7	0.017	0.987
Gender (Male), n(%)	13(100.0)	13(100.0)	/	/
cART treatment (n,%)	6(46.2)	2(15.4)	1.625	0.202
Anti-HBV therapy (n,%)	6(46.2)	2(15.4)	1.625	0.202
CD4^+^ T cell count (cells/μl)	129 ± 33	147 ± 64	0.222	0.827
Log_10_HIV-RNA (mean ± SD)	4.55 ± 0.33	4.61 ± 0.24	0.102	0.919
HBeAg (n,%)	1(7.7)	2(15.4)	0.377	0.539
Log_10_HBV-DNA (mean ± SD)	3.15 ± 0.46	3.24 ± 0.32	0.550	0.587
Blood routine test (mean ± SD)				
White blood cells (10^9^/L)	4.08 ± 0.22	4.22 ± 0.42	0.506	0.617
Hemoglobin (g/L)	110.8 ± 10.5	123.5 ± 15.4	0.726	0.475
Platelets (10^9^/L)	120.5 ± 15.8	127.7 ± 20.6	0.862	0.397
Liver function test (mean ± SD)				
ALT (U/L)	38.6 ± 4.2	40.5 ± 3.7	1.025	0.315
AST (U/L)	38.0 ± 5.0	41.2 ± 6.1	1.002	0.326
Tbil (μmol/L)	11.8 ± 1.9	10.5 ± 2.3	0.908	0.373
Alb(g/L)	38.6 ± 2.8	43.2 ± 3.0	1.321	0.198

SD, standard deviation; cART, Combination antiretroviral therapy; ALT, alanine aminotransferase; AST, aspartate aminotransferase; Tbil, total bilirubin; Alb, albumin.

### Protein identification

**Figure 2:**
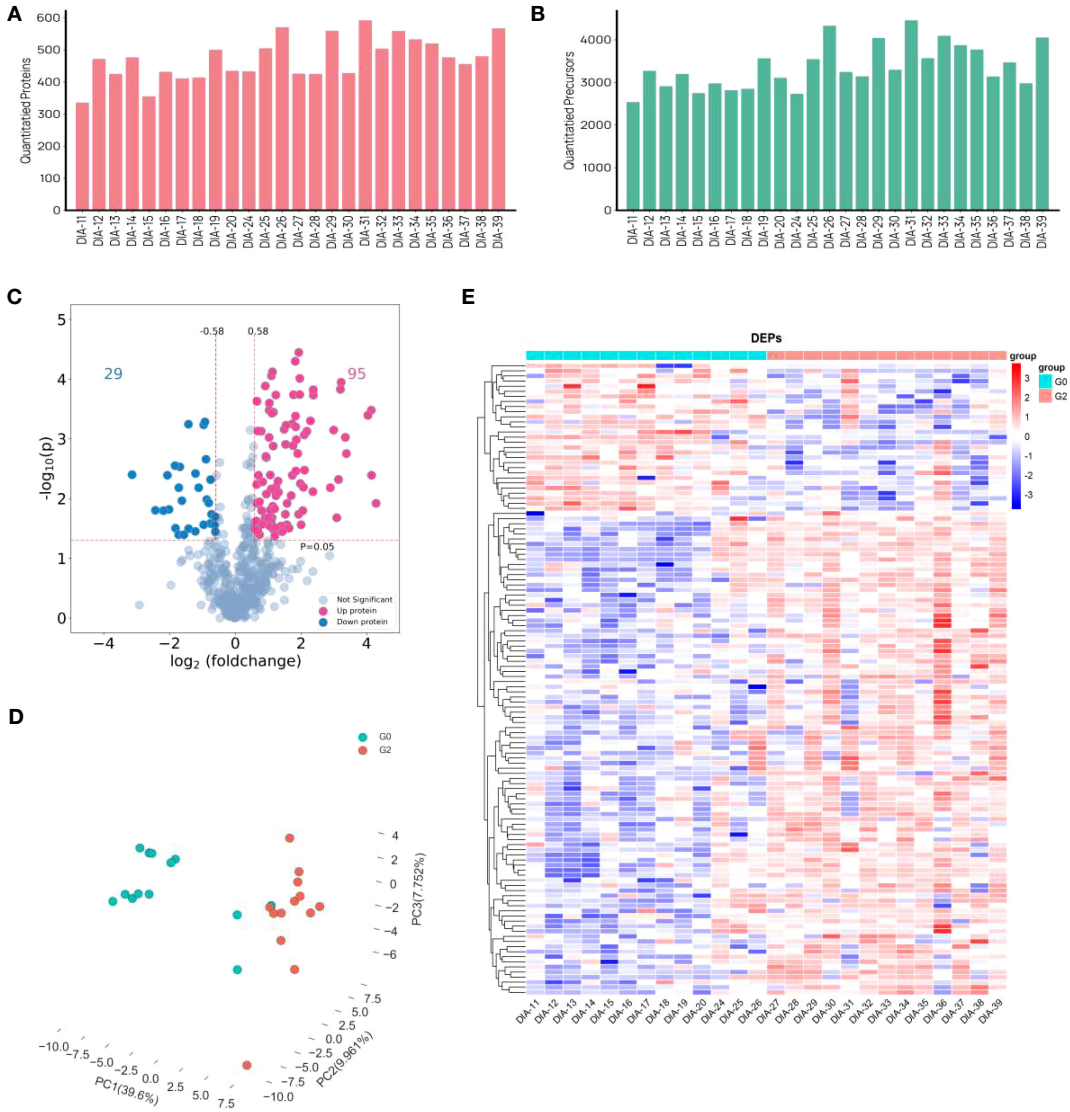
Differently expressed proteins in HCC group and non-HCC group in patients with HIV and HBV co-infection. **(A, B)** The distribution of numbers of quantified **(A)** proteins and **(B)** precursors. **(C)** the volcano plot of DEPs. **(D)** PCA plots. **(E)** Heat map visualization of DEPs.

Using a data-independent acquisition (DIA) technique, plasma proteome profiles were obtained from a total of 26 patients living with HIV and HBV co-infection. Furthermore, 26 proteomes were examined in the entire cohort using a DIA technique, yielding a total of 653 proteins and 8200 precursors (**Figures 2A, B**). 124 proteins were expressed significantly differently in HCC patients than in non-HCC patients. There were 95 proteins that were up-regulated and 29 proteins that were down-regulated (**Figure 2C**). The PCA plot showing the intensities of the 124 differential proteins demonstrated a strong distinction of HCC patients and non-HCC patients (**Figure 2D**). The expression of differential proteins (DEPs) was used to create a heatmap (**Figure 2E**), demonstrating that DEP expression was significantly different between the HCC group and the control group.

### Functional enrichment analysis of differentially expressed proteins

**Figure 3:**
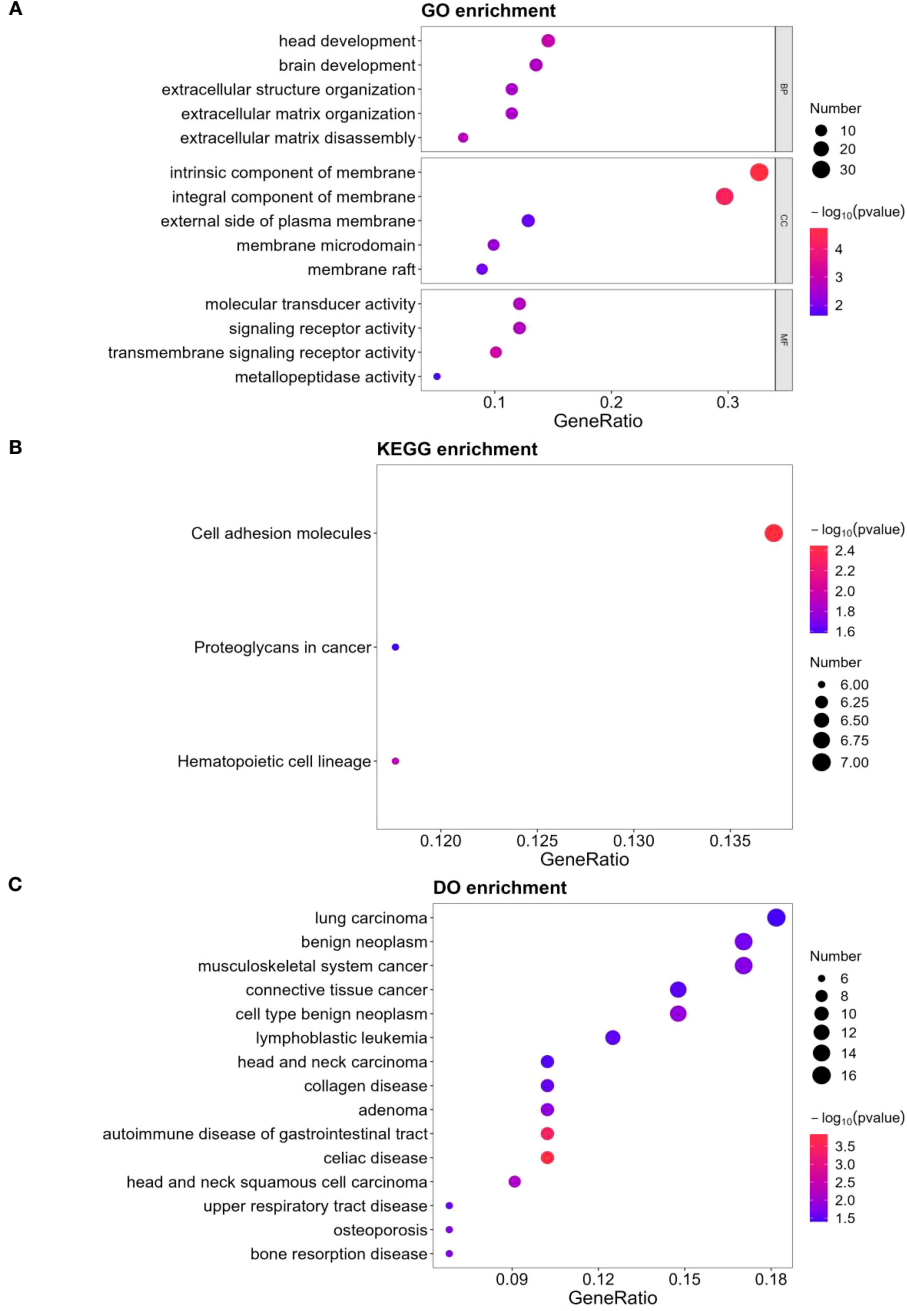
GO, KEGG and DO analysis for 124 DEPs. **(A)** GO-based enrichment analysis of DEPs(two-sided hypergeometric test, *p ≤* 0.05), GO terms were sorted by *p*-value, and top 5 terms of each category were displayed. **(B)** KEGG-based enrichment analysis of DEPs(two-sided hypergeometric test, *p ≤* 0.05), KEGG terms were sorted by *p*-value, and top 3 terms were displayed. **(C)** DO-based enrichment analysis of DEPs(two-sided hypergeometric test, *p ≤* 0.05), DO terms were sorted by *p*-value, and top 15 terms were displayed.

**Figure 3A** depicts the findings of GO keywords with statistical differences in enrichment analysis hypergeometric tests. Three of the five different GO keywords assigned to biological processes were related to extracellular matrix organization. In terms of cellular component, all five enriched terms were membrane components. The top four enriched terms in terms of molecular function were signaling receptor activity, molecular transducer activity, transmembrane signaling receptor activity, and metallopeptidase activity. Proteoglycans were one of the top three DEPs in cancer that were primarily enriched in the KEGG pathway (**Figure 3B**). In terms of DO enrichment analysis, 60.0% of the top 15 enriched terms (shown in **Figure 3C**) were related to various neoplasms.

### Protein-protein interaction network analysis of DEPs

**Figure 4:**
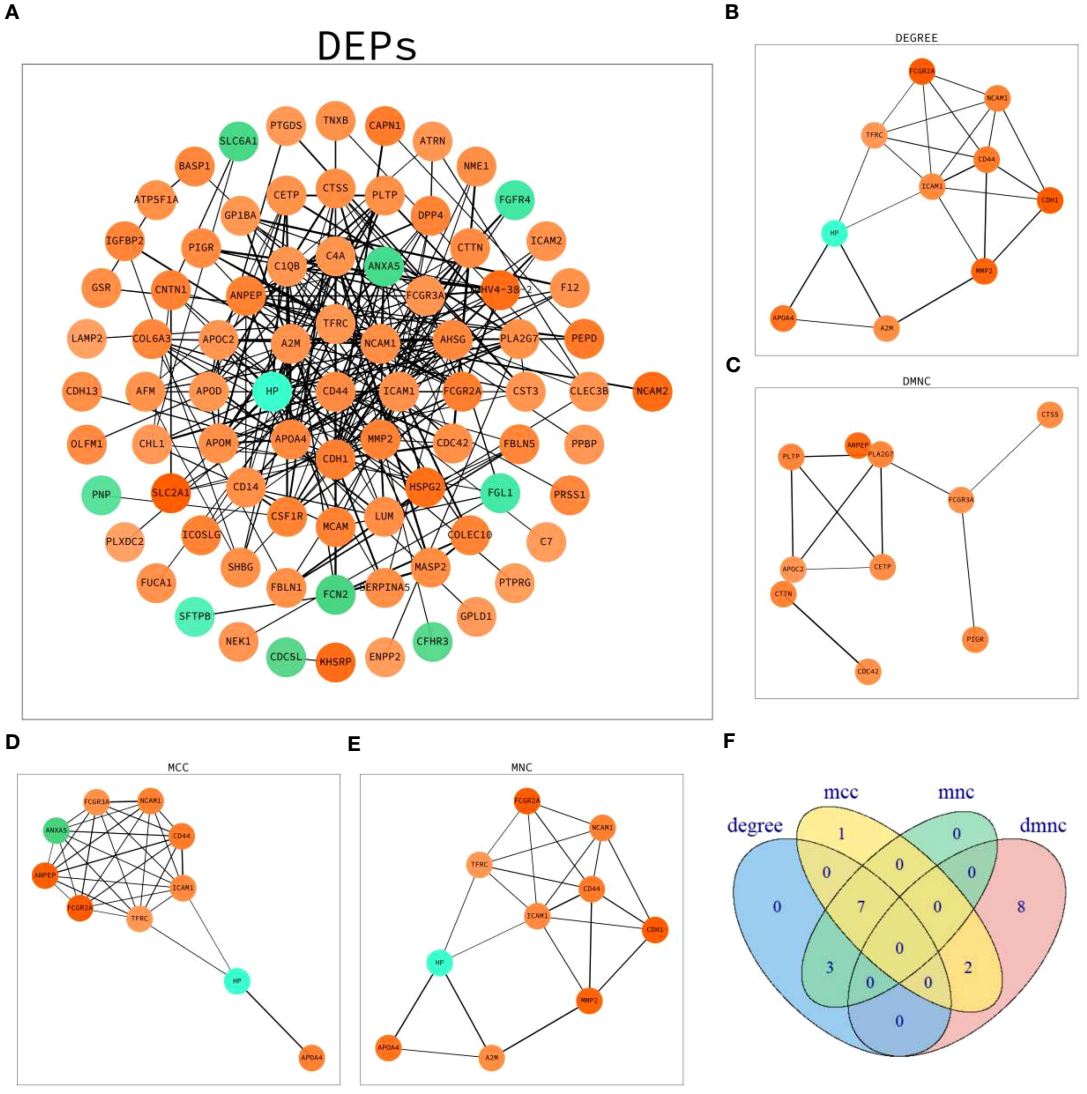
PPI network analysis of DEPs. **(A)** Network graph depicting the correlation of proteins derived from DEPs. **(B)** The hub proteins calculated under DEGREE. **(C)** The hub proteins calculated under DMNC. **(D)** The hub proteins calculated under MCC. **(E)** The hub proteins calculated under MNC. **(F)** Venn diagram based on cross-analysis under four algorithms (DEGREE, DMNC, MCC and MNC). Circles indicated the gene symbol of protein, in which red and green indicated up-regulated and down-regulated proteins, respectively. The thickness of the lines represented the strength of the interaction. The darkness of the colors represented the magnitude of the discrepancy.

Network maps of DEPs with protein–protein interaction (PPI) data from the STRING database were established (**Figure 4A**). The top 10 proteins in four algorithms (DEGREE, DMNC, MCC and MNC) were selected as hub proteins, respectively (**Figures 4B–E**). Moreover, by cross-analyzing and mapping to Venn diagram, CD44 antigen(CD44), intercellular adhesion molecule 1(ICAM1), transferrin receptor protein 1(TFRC), neural cell adhesion molecule 1(NCAM1), apolipoprotein A-IV(APOA4), haptoglobin(HP), low affinity immunoglobulin gamma Fc region receptor II-a(FCGR2A) were identified as hub proteins (**Figure 4F**).

### LASSO regression model

**Figure 5:**
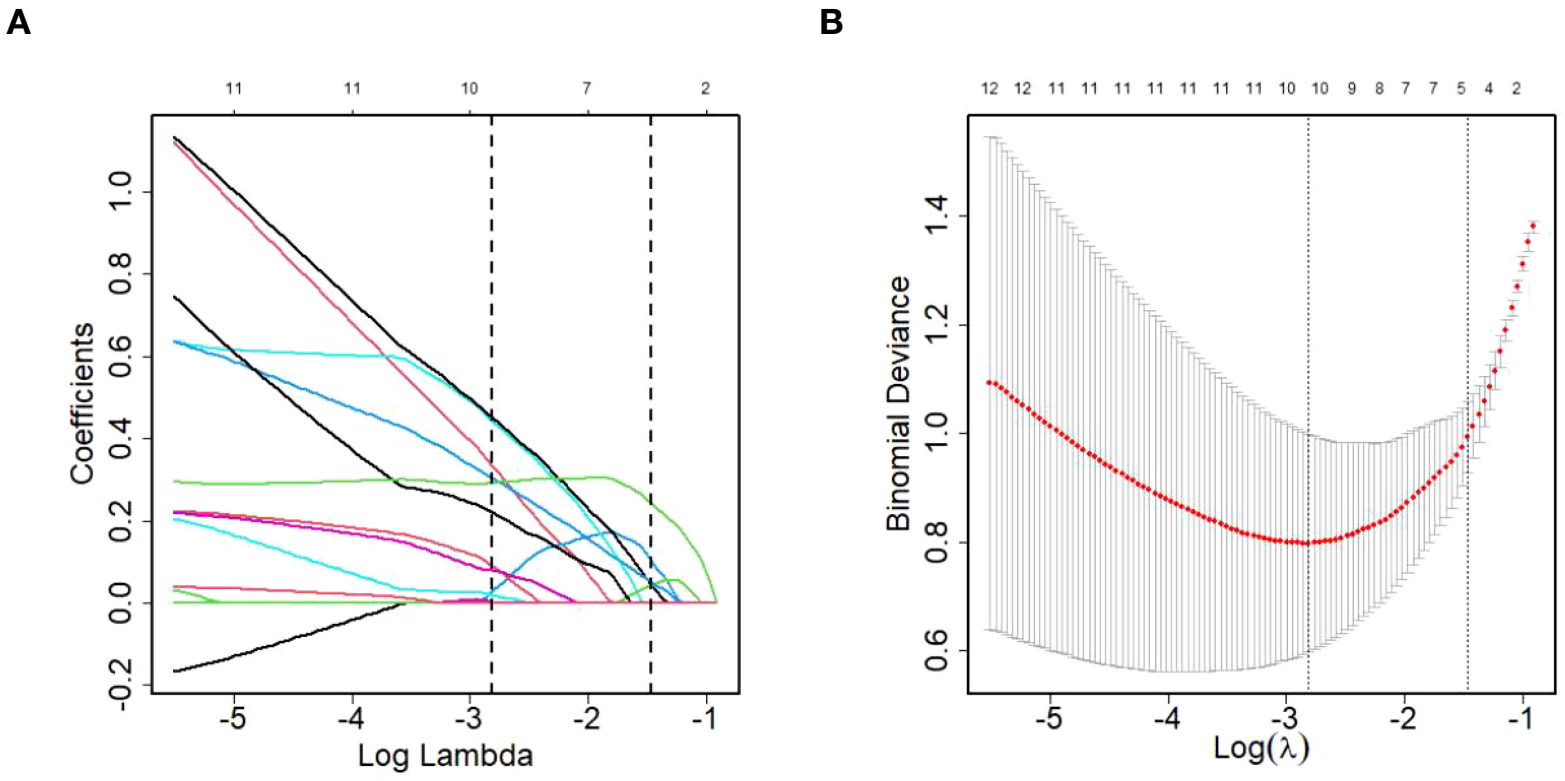
DEPs selection using LASSO regression model. **(A)** LASSO coefficient profiles of the DEPs. **(B)** The relational graph between fitting error and log(λ). Dotted vertical lines were drawn at the optimal values by using the minimum criteria(min criteria) and the 1 standard error of the minimum criteria (1se criteria). λ value was chosen when the fitting error was minimum.

Given the relatively small sample size, the LASSO model was developed to screen the 124 DEPs for core proteins that are closely connected to HCC diagnosis in patients living with HIV and HBV co-infection. Finally, 11 proteins were chosen for additional logistic regression analysis (**Figure 5**). They were golgi-associated plant pathogenesis-related protein 1 (GAPR1), phospholipid transfer protein (PLTP), cytoplasmic junction associated protein 2 (CLASP2), immunoglobulin heavy variable 1-69D (IGHV1-69D), immunoglobulin lambda variable 5-45 (IGLV5-45), alpha-2-macroglobulin (A2M), pantetheinase (VNN1), kallikrein-11 (KLK11), aminopeptidase N (ANPEP), dipeptidyl peptidase 4 (DPP4) and putative hydroxypyruvate isomerase (HYI), and the last five were protease members.

### Model validation

**Figure 6:**
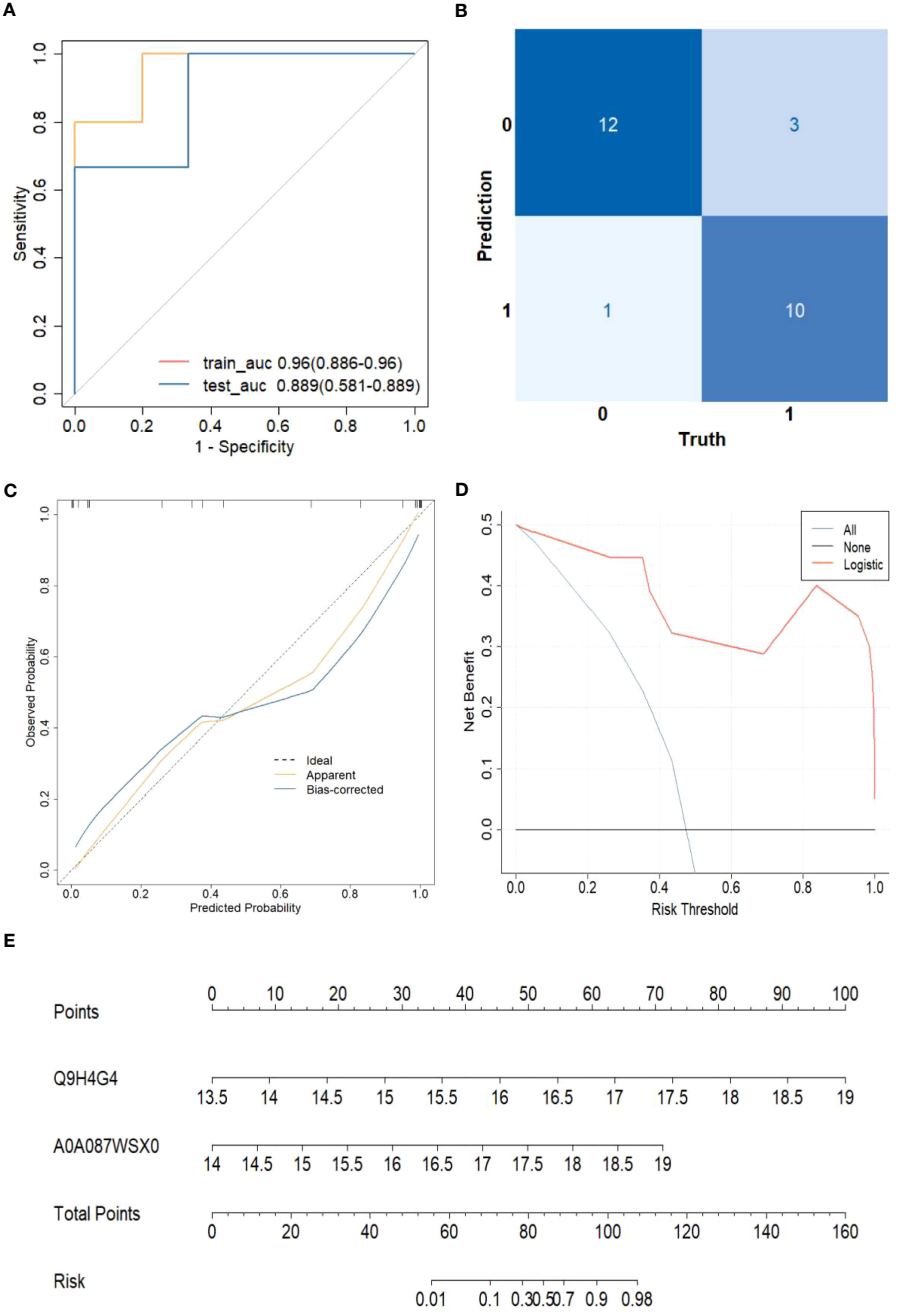
The risk of HCC model evaluation. **(A)** The receiver operating characteristic curve(ROC). **(B)** Confusion matrix. **(C)** Plot depicting the calibration. **(D)** Decision curve analysis curve. **(E)** Nomogram for predicting the risk of liver cirrhosis.

Based on the 11 core proteins screened by the previous part of the LASSO regression model, R programming language was performed for five-cross-validation following the principle of random combination of less than 5 proteins, and the optimal AUC value was used to construct the clinical diagnostic model. Finally, GAPR1 and IGHV1-69D were chosen as the best DEPs combination, and a clinical risk model was built. The model was tested, and the discrimination was satisfactory (**Figure 6A**). The AUC (95% CI) for the train cohort was 0.96 (0.89-0.96) and 0.89 (0.58-0.89) for the test cohort. Furthermore, the findings of these feature combinations’ confusion matrices demonstrated great accuracy for identifying distinct groups (**Figure 6B**). The calibration curve demonstrated that the model performed well when compared to an ideal model (**Figure 6C**). The decision curve analysis revealed favorable results (**Figure 6D**). Using the model data, a nomogram was created (**Figure 6E**).

The sensitivity and specificity of this clinical risk model were 76.9%(10/13) and 92.0%(115/125), respectively. The positive and negative prediction values were 71.4%(10/14) and 97.6%(121/124), respectively.

## Discussion

In HIV/HBV co-infected patients, the incidence rate of hepatocellular carcinoma can reach 2/1000 person-years (21, 22). The tumor microenvironment alters as a result of HCC. According to the findings of this study, 60% of the differential proteins enriched in DO entries are connected with distinct malignancies, implying that different types of tumors have varied pathophysiology but frequently have similar microenvironment modifications. Extracellular matrix (ECM) is a critical component of the tumor microenvironment. Extracellular proteases directly govern and change the tumor microenvironment, as well as indirectly stimulating tumor-cell proliferation, apoptosis, angiogenesis, invasion, and metastasis. In this study, it was found that extracellular matrix organization and membrane component were significantly enriched in GO entries when comparing the differential proteins in the serum of HCC and non-HCC patients, implying that the occurrence and development of HCC can be accompanied by changes in the composition of basement membrane. LG2m, a basement membrane component that is not found in normal tissues, has been shown to be a predictive biomarker for the development of metastatic HCC (23). Elevated serum LG2m is associated with an increased risk of HCC in CHC patients who have achieved sustained virological response. The structural dynamics of basement membrane components in the process of hepatocarcinogenesis remain unknown, and more research is required.

Proteoglycan is an important component of the extracellular matrix, which is linked to the development of atherosclerosis as well as the appearance and spread of malignancies. In this study, we discovered that proteoglycans in cancer are enriched in the KEGG pathway by analyzing biological information from HCC and non-HCC patients. It was discovered that aberrant proteoglycan expression has a significant impact in the formation and progression of HCC.

Impaired hepatic autophagy has been demonstrated to contribute to the pathogenesis of AALD, NAFLD, as well as HBV and HCV hepatitis (24–29), suggesting that deficient autophagy may be a common mechanism for HCC development. Autophagy may also have a paradoxical role in liver cancer, preventing early tumor development while boosting the growth and aggressiveness of already developed liver tumors (30). In mammalian cells, Golgi-associated plant pathogenesis related protein 1 (GAPR-1) can interact with Beclin 1, a major component of the autophagosome nucleation complex, as a negative regulator of autophagy (31). Although the molecular mechanism of this relationship is unknown, GAPR-1 was identified in this investigation as a novel serum biomarker for predicting HCC. The outcome is quite crucial. It was proposed that inhibiting autophagy in combination with other chemotherapeutic medications is a promising therapeutic strategy for HCC.

GAPR-1 interacts with the autophagosome nucleation complex to adversely control autophagy. According to one study, the lipid content influences GAPR-1 binding to biological membranes (32). In this study, phospholipid transfer protein (PLTP) was identified as one of the main DEPs in HCC patients. Through its active form, PLTP performs lipid transfer. The shuttling and redistribution of lipids between lipoproteins, which are ordinarily returned to the liver for metabolism via the reverse cholesterol transport pathway, is thought to be necessary for PLTP-mediated lipid transfer to high-density lipoprotein (HDL). Elevated serum PLTP levels in cancer patients following treatment with topoisomerase I inhibitors may serve as a biomarker for tracking the progression of hypertriglyceridemia (33). We hypothesize that dyslipidemia, via the autophagy effect mediated by GAPR-1, may play a regulatory role in the etiology of HCC.

Furthermore, PLTP has been linked to the diagnostic and anti-cancer actions of specific malignancies. PLTP expression was found to be an appropriate marker for the course and prognosis of gastric cancer (GC) (34). Another study discovered that removing PLTP from U251 cells reduced the expression of epithelial mesenchymal transition markers and invasion molecules matrix metalloproteinases (35). As a result, PLTP was identified as a novel glioma-associated protein that may be involved in the progression of human glioma. In terms of anti-cancer properties, one study found that PLTP is essential in mediating the association of triacyl lipid A with lipoproteins, resulting in an extension of its residence time and magnification of its proinflammatory and anticancer properties (36). Furthermore, PLTP has been identified as a p53 target gene, and forced expression of PLTP reduces colony formation in human tumor cell lines (37). In this study, PLTP was found to be one of the core DEPs in patients with HCC and non-HCC in an HIV/HBV co-infected population, implying that PLTP can be used as a candidate protein to aid in the diagnosis of HCC in this population, and is expected to provide certain clues for the investigation of the mechanism of targeted therapy for HCC.

Five of the 11 key differential proteins identified by the LASSO model were protease members. Vanin-1 (VNN1) was one of the proteases involved. In a prior clinical microarray research, it was first described as a unique clinically derived biomarker of pancreatic cancer-associated new-onset diabetes (38). Another study found that pancreatic cancer cells overexpressing VNN1 aggravate paraneoplastic islet dysfunction by causing oxidative stress and β-cell dedifferentiation (39). Pantetheinase VNN1 was discovered to be expressed differentially in HCC and non-HCC individuals, providing evidence to support the function of oxidative stress in HCC.

Kallikrein-11 (KLK11) was the second protease. The KLK gene family is the largest protease group in the human genome. KLK11 was first isolated from the human hippocampus and was found to cleave fewer substrates than other types of proteases, indicating a high degree of substrate specificity (40). KLK11 is thought to be a prostate, ovarian, and breast cancer biomarker (41–43). According to one study, KLK11 mRNA expression is a unique and independent biomarker in laryngeal cancer that can be used for diagnostic and prognostic purposes (44). Another study found that Kallikrein 11 was one of the contributing factors implicated in the rise of CAF-mediated oral squamous cell carcinoma invasion (45). According to one study, changes in KLK11 expression levels can be utilized as additional data to predict clinical outcome and prognosis in breast cancer, gastric cancer (46), and low rectal cancer (47). A Kaplan-Meier survival curve study demonstrated that elevated KLK11 expression was linked to a poor prognosis of cholangiocarcinoma (48). Stronger Kallikrein-11 expression appears to be associated with improved survival rates in non-small cell lung cancer (49). Although the preceding research established that KLK11 is associated with tumor diagnosis and prognosis, no studies have reported a link between KLK11 and HCC. The findings of this study help to expand the database of KLK11 and tumor associations.

Aminopeptidase N (APN, commonly known as CD13) was the third protease. It is a transmembrane protein that is widely expressed. APN was commonly elevated in HCC tumor tissues and cell lines with strong metastatic potential (50). It has a distinct canalicular pattern when expressed in normal and neoplastic liver tissue (CD13(can)). CD13(can) has been shown to be more sensitive than AFP in distinguishing between HCC and non-HCC (51). APN is engaged in numerous sorts of tumor cellular processes and is a possible anti-cancer therapeutic target. According to the findings of one study, APN causes BCKDKS31 phosphorylation and activates its downstream pathway to enhance HCC proliferation and metastasis (50). The new APN inhibitors 4cc and 24F were shown to be anti-cancer agents for HCC by activating mitochondrial apoptosis pathways and inhibiting HCC cell invasion and angiogenesis (52, 53). Furthermore, CD13 is likely to be a promising target for reducing HCC resistance (54). These findings imply that aminopeptidase N (APN)/CD13 is linked to HCC, and our work provides information on the difference in serum APN expression between HCC patients and non-HCC patients in an HIV/HBV co-infected community. It is proposed that APN/CD13 may be a candidate noninvasive serological marker to aid in the diagnosis of HCC.

Dipeptidyl peptidase 4 (DPP4) was the fourth protease. It has pro-apoptotic effects as a regulatory protease. It has been discovered to be improperly regulated in patients with HCC, similar to the findings in this investigation (55–57). As a result, its therapeutic importance is worth summarizing and delving into. According to one study, the DPP4 gene family’s mRNA expression is substantially increased in human HCC and is related with poor survival in HCC patients (58). As a result, targeting the DPP4 enzyme family could be a unique and effective strategy for promoting anti-tumor immunity in HCC. DPP-4 inhibitors were observed to be related with a decreased incidence of HCC in individuals with Type 2 Diabetes Mellitus (DM) with chronic HCV or HBV infection in two Taiwanese studies (59, 60). A mouse investigation revealed that DPP4 suppresses HCC growth in addition to its anti-diabetic properties (61). It is worthwhile to study the association between the usage of DPP4 inhibitors and the occurrence of HCC in HIV/HBV co-infected individuals.

Notably, the expression of putative hydroxypyruvate isomerase (HYI), the fifth protease, was shown to be elevated in HCC patients. The current understanding of HYI is rather limited. So yet, we have not retrieved studies on HYI. However, our findings imply that HYI is a new protease linked to HCC.

The findings of this investigation in an HIV-infected population revealed that cytoplasmic junction associated protein 2 (CLASP2) was differently upregulated in HCC. According to the research, HIV uses a variety of specific microtubule plus-end tracking proteins to drive the production of stable microtubules shortly after virus entrance and boost the early phases of infection (62). CLASP2 has been discovered as a host component that allows HIV to induce microtubule stability and boost early infection in natural target cell types (63). We hypothesized that the oncogenesis and process of HIV/HBV co-infection in HCC patients are exceedingly complex, and CLASP2 is one of the numerous pathogenic elements of HCC. This could be the difference between the factors impacting HCC in the PLWH group and the non-HIV infected population.

A mass spectrometry analysis discovered that IGLV5-45, located in the variable region of the immunoglobulin Lambda light chain, is the light chain peptide with the greatest variation between active and stable SLE (63). It was found to be associated with anhedonia/retardation (64). In HCC patients, the expression of immunoglobulin lambda variable 5-45 (IGLV5-45) and immunoglobulin heavy variable 1-69D (IGHV1-69D) in serum was enhanced, indicating B cell hyperactivity and an inflammatory condition. The immunoglobulin lambda light chain variable region, which is strongly associated to antigenantibody interactions and therapeutic target development, gave rise to IGLV5-45 molecules. It was identified as DEPs, indicating that HCC progression was tightly linked to immune response.

We acknowledge that there are some limitations with this study. First, since all of the participants were male, the results of this study are limited to a certain extent, and whether the results are applicable to female patients requires more examination. Second, to strengthen and promote the application value of predictive indicators of HCC, validation study would be done in a relatively larger population, including both HBV mono‐infected patients and HIV/HBV co‐infected patients. Third, the sample size of this study is relatively small. In the future, conditions will be created to supplement appropriate samples to further improve the experimental results and analysis. However, despite these limitations, the bio‐informatics analysis results in this study could provide valuable clues for future study about early diagnosis and pathogenesis of HCC.

## Conclusions

This is a proteomics-based study in an HIV/HBV co-infected group. A total of 124 DEP signatures were detected in patients with and without HCC, with 95 DEPs being up-regulated and 29 DEPs being down-regulated. The functional examination of DEPs indicates that the majority of them are abundant in extracellular matrix organization and membrane components that comprise the tumor microenvironment. Proteoglycan is a component of the extracellular matrix that is abundant in the KEGG pathways in HCC condition. The LASSO model screened eleven core differential proteins, including GAPR1, PLTP, CLASP2, IGHV1-69D, IGLV5-45, A2M, VNN1, KLK11, ANPEP, DPP4 and HYI, and the last five belonged to protease members. The findings could aid in the discovery of novel biomarkers for HCC diagnosis in patients living with HIV and HBV co-infection. Further research into the molecular processes underpinning how these DEPs influence HCC formation and progression may aid in the development of better therapeutic options for HCC prevention and therapy.

## Data availability statement

The data presented in the study are deposited in the ProteomeXchange Consortium via the iProX repository, accession number PXD046369. IPX0007140003 in PXD046369 is the original sequence information of this study.

## Ethics statement

The studies involving humans were approved by the institutional review board of the Zhongnan Hospital of Wuhan University. The studies were conducted in accordance with the local legislation and institutional requirements. Written informed consent for participation in this study was provided by the participants’ legal guardians/next of kin.

## Author contributions

HK: Conceptualization, Formal Analysis, Methodology, Validation, Writing – original draft, Writing – review & editing. RY: Conceptualization, Data curation, Investigation, Methodology, Software, Validation, Writing – original draft, Writing – review & editing. HL: Data curation, Resources, Software, Writing – original draft, Writing – review & editing. ML: Conceptualization, Formal Analysis, Methodology, Supervision, Writing – original draft, Writing – review & editing. HH: Conceptualization, Formal Analysis, Resources, Software, Writing – review & editing. EZ: Conceptualization, Data curation, Investigation, Methodology, Resources, Supervision, Writing – review & editing. KZ: Investigation, Methodology, Resources, Writing – review & editing. YY: Methodology, Resources, Software, Visualization, Writing – review & editing. RRY: Conceptualization, Formal Analysis, Funding acquisition, Investigation, Methodology, Project administration, Software, Supervision, Validation, Visualization, Writing – original draft, Writing – review & editing.
